# Supplementary material for: A high-resolution mRNA expression time course of embryonic development in zebrafish
Source: eLife. 2017 Nov 16;6:e30860. doi: 10.7554/eLife.30860 (PMC5690287; doi:10.7554/eLife.30860)
Supplement: Supplementary file 6. [file elife-30860-supp6.zip › biolayout-clusters-files/Cluster092-genes.html]

Cluster092


# Cluster092: Genes

| Ensembl ID | Gene Name | Chr | Start | End | Biotype |
| --- | --- | --- | --- | --- | --- |
| ENSDARG00000057830 | dnmt3bb.2 | 23 | 17035036 | 17078731 | protein\_coding |
| ENSDARG00000011190 | fgfr1b | 10 | 42761087 | 42833263 | protein\_coding |
| ENSDARG00000070957 | im:7160594 | 24 | 39306367 | 39314952 | protein\_coding |
| ENSDARG00000034610 | insb | 14 | 28257123 | 28262194 | protein\_coding |
| ENSDARG00000011246 | krr1 | 4 | 2409622 | 2415958 | protein\_coding |
| ENSDARG00000088346 | miip | 11 | 40240896 | 40255741 | protein\_coding |
| ENSDARG00000012513 | sdcbp2 | 23 | 16722278 | 16755843 | protein\_coding |
| ENSDARG00000008188 | sf1 | 7 | 22538748 | 22549659 | protein\_coding |
| ENSDARG00000104178 | si:ch73-152o7.1 | 3 | 7937108 | 7948666 | protein\_coding |
| ENSDARG00000056954 | si:dkeyp-38g8.5 | 4 | 16326716 | 16330786 | protein\_coding |
| ENSDARG00000002970 | ss18 | 2 | 2235842 | 2252260 | protein\_coding |
| ENSDARG00000098228 | thrap3b | 19 | 3934445 | 3956165 | protein\_coding |
| ENSDARG00000077307 | tmem201 | 23 | 29900593 | 29931546 | protein\_coding |
| ENSDARG00000007863 | tra2a | 19 | 20593564 | 20606487 | protein\_coding |
